# Keep-or-drop multidimensional control systems in professional organisations: evidence on the use of the balanced scorecard in healthcare

**DOI:** 10.1108/JHOM-09-2023-0287

**Published:** 2024-04-11

**Authors:** Anna Prenestini, Stefano Calciolari, Arianna Rota

**Affiliations:** Department of Economics, Management and Quantitative Methods, Università degli Studi di Milano, Milan, Italy; Department of Economics, Management, and Statistics, Università degli Studi di Milano-Bicocca, Milan, Italy; Università degli Studi di Milano, Milan, Italy

**Keywords:** Performance, Balanced scorecard, Health care, Management control systems, Strategy execution

## Abstract

**Purpose:**

During the 1990s, Italian healthcare organisations (HOs) underwent a process of corporatisation, and the most innovative HOs introduced the balanced scorecard (BSC) to address the need for broader accountability. Currently, there is a limited understanding of the dynamics and outcomes of such a process. Therefore, this study aims to explore whether the BSC is still considered an effective performance management tool and analyse the factors driving and hindering its evolution and endurance in public and non-profit HOs.

**Design/methodology/approach:**

We conducted a retrospective longitudinal analysis of two pioneering cases in the adoption of the BSC: one in a public hospital and the other in a non-profit hospital. Data collection relied on accessing institutional documents and reports from the early 2000s to the present, as well as conducting semi-structured interviews with the internal sponsors of the BSC.

**Findings:**

We found evidence of three main categories of factors that trigger or hinder the adoption and development of the BSC: (1) the role of the internal sponsor and professionals’ commitment; (2) information technology and the controller’s technological skills; and (3) the relationship between the management and professionalism logics during the implementation process. At the same time, there is no evidence to suggest that specific technical features of the BSC influence its endurance.

**Originality/value:**

The paper contributes to the debate on the key factors for implementing and sustaining multidimensional control systems in professional organisations. It emphasises the importance of knowledge-based assets and distinctive internal capabilities for the success of the business. The implications of the BSC legacy are discussed, along with future developments of multidimensional control tools aimed at supporting strategy execution.

## Introduction

Performance measurement and management systems and tools of any organisations have traditionally focused on their financial accounting indicators. Since the 1980s, scholars have argued that such a control focus is problematic for managing complex and ever-changing organisations (Niven, 2008; Simons, 2014) because it disregards organisations’ intangible assets (e.g. distinctive know-how, customer loyalty and employees’ skills) despite their competitive relevance (Kaplan and Norton, 2004). In particular, financial measures are typically backward-looking, while metrics concerning intangible assets are forward-looking and indicate expected performance trajectories (Kaplan and Norton, 2001a), supporting strategy execution.

This line of argument is even stronger when considering highly professionalised organisations, where intangible assets are generally emphasised. Specifically, healthcare organisations (HOs) are brain-intensive entities where intellectual capital and knowledge-based assets are crucial in most operational and strategic processes (Lega, 2008; Scott, 1965; Scott and Backman, 1990). In addition, the healthcare industry operates in an extremely dynamic environment, with constantly evolving technology, medicine, epidemiological conditions, policies and regulatory frameworks.

In this context, healthcare managers need managerial tools to facilitate communication and strategy execution, monitor strategic objectives and rapidly adapt the strategy to contingent environmental changes (Prenestini, 2008). However, the concept of control is challenged by the inherent “latent conflict” between the health professionals’ values and objectives and the organisational/managerial values and goals (Lemieux-Charles *et al*., 2003; Prenestini *et al*., 2021). In particular, the logic of professionalism embeds values such as concern for individual health outcomes, preservation of clinical autonomy and the primacy of medical specialty, while the logic of management focuses on strategies that target populations/groups and foster efficiency in service delivery (Garelick and Fagin, 2005). The resulting conflict nurtures misunderstanding or uncertainty about the true purpose of control and the actual way to implement it without interfering with health outcomes. Consequently, professionals’ resistance along with the weakness of the management control function result in the delay in implementing effective control systems in the healthcare sector compared to other industries (del Vecchio, 2000). In the case of hybrid roles, some authors claim that the traditional cultural reluctance to engage in performance management practices is also attributed to the lack of specific training and role development activities (Giacomelli, 2020).

Such tension is particularly high for organisations operating in health systems with a dominant public actor, such as the Italian National Health Service (NHS). In these systems, performance is articulated in several dimensions to prevent the financial perspective from overshadowing other institutional and political priorities (Prenestini, 2008). Since 1992, the Italian NHS has undergone significant decentralisation and corporatisation due to the New Public Management (NPM) wave. This process introduced important discontinuities, including prospective payment systems and performance-based accountability systems. Consequently, management control systems became an important means to meet the objectives of such reforms (Prenestini, 2008). In the early 1990s, the design and implementation of performance management systems aimed to improve financial results and outputs, such as the volume of service production. However, such a narrow approach to performance management showed several weaknesses. In response, the significance of measuring performance across multiple dimensions with a comprehensive range of metrics became increasingly important in the 2000s (Vainieri *et al*., 2020).

The balanced scorecard (BSC) is considered an effective control framework for managing such tension and developing a multidimensional control tool. It has been implemented in HOs in the 30 years following when it was conceived in the early 1990ss (Bohm *et al*., 2021). In particular, the BSC had been used as an internal control tool by several Italian HOs during the early 2000s (Baraldi, 2005; Gonzalez-Sanchez *et al*., 2018). However, later, several HOs dismissed the BSC, and interest in the tool declined. According to Baraldi (2005), in 2004 some “pioneer” hospitals (16.8% of about 230 respondents) were engaged in the early stages of BSC development. By 2009, only 11% of the 184 respondents reported using the BSC, and 13% were committed to implementing the tool, while 9% had initially implemented the BSC but later abandoned the project (Cerismas, 2009).

Few studies have performed longitudinal analyses of the evolution of the BSC implementation process. Among others, Bassani *et al*. (2022) used a prospective approach that focused on the dynamic use of the BSC through a case study of an Italian public hospital, which was analysed in 2013 and 2018. However, to the best of our knowledge, no previous study has used the longitudinal approach that we propose to investigate the reasons for the persistence of the use or dismissal of the BSC in HOs. Moreover, Betto *et al.* (2022) recently called for further research to converge on the barriers and drivers of BSC implementation.

Our study has a twofold objective: (1) to analyse the long-term implementation process of the BSC in two HOs, with a specific focus on how each organisation has addressed the conflict between professional and managerial logics and (2) to identify the conditions that may trigger its persistence or dismissal.

First, we elaborate on a theoretical background section regarding the characteristics of the BSC and its application to healthcare. Next, we describe the data and methods used in the study. The two sections following the methodology focus on results and discussion, with the former elaborated in a chronological fashion (to highlight the longitudinal evolution of each case) and the latter articulated conceptually to clearly identify barriers/drivers of BSC implementation. Finally, we present our conclusions and discuss the practical implications.

## Theoretical background

The BSC, conceived by Kaplan and Norton (1992), is a multidimensional control framework that facilitates the execution of strategy (Kaplan and Norton, 1996, 2001a). The framework relies on the underlying causal paths between different perspectives. In general, intangible resources and employee efforts (from the learning and growth perspective) enhance internal processes, improve customer satisfaction and ultimately lead to the generation of economic value (from the financial perspective). Each perspective can be expressed in key performance areas (KPAs) associated with key performance indicators (KPIs). This approach supports the implementation and communication of the corporate strategy across the organisation (Kaplan and Norton, 2004; Othman, 2006).

The BSC implementation can accommodate a variety of contexts and contingencies (Frittoli and Mancini, 2004; Zelman *et al*., 2003). In this regard, Norton and Kaplan proposed adaptations of the BSC’s KPAs for public and non-profit organisations. Several other authors have adapted the BSC for HOs (Aidemark, 2001; Curtright *et al*., 2000; Inamdar *et al*., 2002; Urrutia and Eriksen, 2005; Zelman *et al*., 2003), including customising the tool for use in academic medical centres (Catuogno *et al*., 2017; Trotta *et al*., 2013; Zelman *et al*., 1999). In healthcare, BSC implementation often involves a redesign of the original framework, affecting both the number and types of perspectives (Bohm *et al*., 2021; Gonzalez-Sanchez *et al*., 2018).

At the implementation level, the BSC must be based on a clear, shared strategy (Bassani *et al*., 2022) and should incorporate data-intensive, inspiring strategies into daily work tasks (Frittoli and Mancini, 2004). Health professionals may use the BSC as a compass to guide their practices and as a learning tool aimed at understanding the relationships between clinical and financial governance (Baraldi, 2005).

Following Henri’s (2006) framework, Bassani *et al*. (2022) demonstrated that the function of the BSC may shift from an attention-focusing role to a monitoring and a legitimising role.

The conceptual framework of this study is based on the socio-technical theory (STT), which views organisations as consisting of two independent but correlated and interactive systems: the social and the technical (Bostrom and Heinen, 1977a). The social system encompasses the attributes of individuals (e.g. attitudes, skills, values and beliefs), their relationships with others and their interactions with the organisational structure. The technical system refers to processes, tasks and technology designed to perform work activities (Bostrom and Heinen, 1977a; Malatji *et al*., 2020).

The framework guided our analysis of the implementation of the BSC in each case. Specifically, the technical component of the framework includes analysing the methods and tools necessary for implementing the BSC, as well as potential changes in work tasks and the introduction of new technological assets (Bostrom and Heinen, 1977a, b; Oesterreich *et al*., 2022). The social dimension considers the human capital and organisational culture traits associated with the implementation and use of the new tools. Furthermore, due to the highly regulated nature of the healthcare industry, we explicitly examined the main environmental changes that could have influenced the social and technical systems.

## Research methods and data

Our study aims to investigate complex organisational and institutional processes. Therefore, we chose a method designed to answer the “how?” and “why?” of the phenomenon under investigation, suitable for the exploratory nature of our study. This method was designed to generate hypotheses and, eventually, preliminary theories grounded in the detailed results. Additionally, the evolving dynamics of the analysed business environment (e.g. regulatory changes, advancements in medical technology and ever-changing practices) may undermine rigid explanatory models regarding managerial control. In an empirical setting, a case study represents a method that allows the study of a phenomenon within its real-life context (Yin, 2018).

First, our research focused on the Italian NHS as the inclusion criteria. Based on the reviewed Italian scientific literature, we identified the most suitable case studies for our research. Secondly, we selected case studies from the pool of cases discussed in Baraldi’s (2005) work and in the literature review by Gonzalez-Sanchez *et al*. (2018). We performed the selection by defining specific exclusion criteria: (1) multidimensional control systems not directly classifiable as the BSC; (2) BSC applied to regional healthcare systems or focused on specific specialty units; (3) HOs that no longer exist due to mergers and/or changes in their institutional form and (4) key informants who are no longer available for in-depth interviews. The exclusion criteria were designed to foster comparability and ensure the longitudinal nature of the analysis.

The application of the defined criteria led us to select two case studies: one in San Martino University Hospital (Research Hospital 1, RH1) in Genoa and another in a private non-profit research hospital accredited with the NHS (Research Hospital 2, RH2) located in Northern Italy. These two HOs were pioneers in adopting the BSC in the early 2000s.

The data collection relied on access to several institutional documents and reports related to the BSC from its inception to the present. These included minutes of the main project management meetings, BSC reports and forms, integrated annual reports, official documents on the performance management system and press releases. Furthermore, we administered semi-structured interviews with the main sponsors for introducing and developing the control tool, including the director of the Management Control Unit (MCU) at RH1 and the former Chief Executive Officer (CEO) at RH2.

The researchers analysed the two case studies using a conceptual framework and guidelines for semi-structured interviews grounded in the socio-technical approach (Bostrom and Heinen, 1977a; Malatji *et al*., 2020) and in the relevant literature concerning the BSC in healthcare (Aidemark, 2001; Aidemark and Funck, 2009; Dyball *et al*., 2011). The guidelines were articulated into four conceptual areas: (1) the evolution of the main technical and conceptual features of the BSC since its introduction; (2) the role of the main sponsor in the BSC implementation and development; (3) internal and external drivers/barriers for the evolution of the tool and its eventual dismissal and (4) performance management logic underlying the use of the BSC. We also draw upon existing literature (Baraldi, 2005) to customise the guidelines based on the established elements related to the BSC’s development in each selected organisation.

Therefore, we primarily followed a deductive approach, as the interviews were conducted based on a predefined guide rooted in the existing literature. However, we flexibly managed the interviews to preserve the flow of the discussion and capture all the tangible and intangible elements that could be involved in the development or dismissal of the tool.

We validated the preliminary framework and the interview guidelines with the assistance of a key expert in the analysis and development of BSC in healthcare. The expert is a former researcher at a leading Italian research centre on healthcare management and has been involved in several action research initiatives for the implementation of BSC in HOs within the Italian NHS. The expert opinion was important for triangulating the relevance and accuracy of the conceptual areas of investigation identified in the interview guidelines (Miles and Huberman, 2014) and including any further missing aspects worth investigating.

The researchers shared the guidelines with the interviewees a few days before the scheduled date to facilitate the collection of useful material when needed. The interviews were carried out using the Microsoft Teams platform between May 27 and June 9, 2021, by a senior and a young researcher (AP and AR, respectively). On average, each interview lasted for two hours. At the end of each interview, the researchers asked whether the interviewee had any further relevant elements to add. This allowed us to include aspects that were not directly addressed by the questions. The interviews were type-recorded to facilitate the ex-post transcription and further systematisation of the data (Miles and Huberman, 2014). Interview transcriptions, field note write-ups and institutional documents were coded separately by the researchers in two rounds. The second round was used to standardise and incorporate any new relevant codes that emerged during the first round. After completing the initial work, we contacted the interviewees to conduct a one-hour follow-up interview for each case. This allowed us to address any gaps in information or uncertainty and to ensure that we had fully comprehended the phenomenon under investigation.

The further analysis enabled the researchers to identify patterns that were useful in addressing the initial research questions and explaining the phenomenon under study. The complete process facilitated an in-depth understanding of the phenomenon for explanatory/interpretative purposes and allowed the researchers to revisit the data in cases of interpretative conflict or conceptual inaccuracy (Miles and Huberman, 2014).

Finally, the analysis progressed to the discussion phase, aimed at explaining the significance of the data collected in light of existing knowledge about the phenomenon.

## Results

### Case study 1: public research hospital (RH1)

RH1 is the largest general public hospital in the Liguria Region, recognised as a Scientific Institute for Research and Treatment (Istituto di Ricerca e Cura a Carattere Scientifico, IRCCS) for oncology and neurosciences by the Ministry of Health. The RH1’s mission consists of achieving excellence in biomedical research and health services management and organisation, innovation in care and knowledge transfer models and high performance in clinical practices for inpatients.

In the early 2000s, RH1 implemented the BSC mainly due to two internal factors: a) the development of information technologies for performance reporting and b) the need to consolidate different measures building the corporate budget (such as activity volume, expenditure availability, material consumption and indicators of efficiency and appropriateness).

In 2002, the management control system introduced a new reporting system with a high degree of automation and transparency of data and information for budget definition and overall performance assessment. The system has been developed to encompass all aspects of management, production and cost, providing summary views (at both the departmental and organisational levels) and detailed views issued every 10 days.

In 2004, the RH1 launched the first version of the BSC, called the “Budget Scorecard”. Initially, it was an experimental tool (named the “Traffic Light Card” because of its iconography) linked to budget objectives and used by the MCU to develop customised indicators that are helpful for end users in estimating trends and congruity with the activities of any specialty unit.

In this first phase, the design and development of the BSC were driven by the principles of simplicity, pertinence with the company’s macro-objectives, continuity, coherence, integration of performance measures and uniformity of standards and formulas. Based on these criteria, the scorecard was configured with four perspectives: *production, costs, efficiency, organisation* and *quality*. For each perspective, specific objectives were identified to express the company’s strategic macro-objectives. For each goal, one or more indicators were identified. The traditional perspectives were modified to adopt labels that health professionals easily accepted and indicators that were perceived as useful. However, the “costs” perspective aligns with the traditional “financial” perspective, the “production” perspective to “internal processes” and the “efficiency” and “organisation and quality” perspective to “learning and growth”. Interestingly, the traditional customer-oriented perspective is absent here.

The next step of the project consisted of automating the scorecards by creating a computer-based management dashboard. The chosen information technology (IT) system was strategic performance management (SPM), which enables the customisation of the dashboard through a user-friendly interface.

In 2011, the BSC became the official tool for evaluating the organisational performance of the RH1’s organisational units. Therefore, the BSC has linked performance evaluation with budget incentives since 2012.

The 2012 revision of the BSC further modified its perspectives (*resources, activity, organisation, quality, safety and risk control and research*) to better adapt the tool to the reformed healthcare context. A major institutional change occurred in 2011 when the RH1 merged with the Cancer Institute, acquiring the status of a research hospital (IRCCS) for oncology and neuroscience. Consequently, the management decided to add a fifth perspective dedicated to research. The analysis of this perspective was supported by a data warehouse developed in-house by the MCU.

The newly introduced “resource” perspective, which includes objectives related to the budget allocated for consumables and human resources management, represents an evolution from the previous “costs” perspective. The “activity” perspective, which encompasses the objectives of service volumes, appropriateness and efficiency, signifies a reinterpretation of the earlier “production” and “efficiency” perspectives. The “organisation” perspective concerns the use of economically relevant resources (e.g. beds, operating rooms and expensive drugs), training objectives and the timely completion of hospital discharge forms. The “quality, safety and risk control” perspective regards the results of internal audits about safety and quality, the completeness and accuracy of healthcare documentation and the minimisation of sentinel events (e.g. repeated hospitalisations, falls and bedsores). The last two perspectives represented a reinterpretation of the previous “organisation and quality” perspective. The “research” perspective suggests focusing on a performance area linked to the new institutional status of RH1 as an IRCCS. Still, there is no evidence of the traditional “client” perspective.

Since the COVID-19 pandemic disrupted the traditional managerial accounting cycle in 2020, the BSC has undergone another change, with the latest version released on 26 May 2021. The “appropriateness”, “effectiveness” and “efficiency” perspectives were enumerated from the previous perspectives. The new version of BSC is characterised by increased customisation of the reporting outputs, allowing the production of “navigable” spreadsheets for each specialty unit. The first section of the new BSC reports is contingent on the clinical category to which the specialty unit belongs. It includes indicators for various categories such as surgery, medicine, service (without inpatient beds), intensive care units, emergency units and technical-administrative units. The second section of the report shows the specific objectives of each specialty unit based on five external institutional sources: (1) the “System of Assurance of Essential Levels of Care”; (2) the “National Outcomes Plan” (Piano Nazionale Esiti); (3) the “MES indicators” by Sant’Anna School of Advanced Studies, Pisa; (4) the “National Waiting List Management Project”; and (5) the “Oriented Production Project” (consisting of specific production targets defined by the Liguria Region to recover passive mobility). In the last section, the MCU periodically identifies the strengths and weaknesses of each specialty unit and assigns specific performance targets.

Each organisational unit receives a dynamic sheet containing only its own objectives and three to four customised additional objectives that have been previously agreed upon with their evaluators. The MCU provides the specialty units with all available corporate and regional benchmarks.

Strategy maps have never been adopted in RH1, but the MCU has always used implicit cause-and-effect correlations to pursue cross-objectives. For example, the same objectives and KPIs are included in the scorecards of different organisational units, such as cost-cutting in diagnostics for the laboratory and reduction of diagnostic costs per Diagnosis Related Groups (DRG) in clinical units. The rationale consisted of fostering cross-unit collaboration.

In RH1, the MCU has had the same director in charge since 2002, and he has been the internal sponsor since the introduction of this tool.

The director of the MCU has been entrusted with the strategic apex since the beginning of the implementation and was delegated by top management to allocate resources between departments and organisational units.

The MCU has acquired the skills to develop and manage the BSC dashboard and databases autonomously. Moreover, the MCU is committed to introducing improvements of technological tools annually, as well as training the organisational units in their effective use.

If we had not developed the IT procedures for the BSC internally in the MCU, it would not have been possible to achieve the results obtained (Director of the MCU).

### Case study 2: private non-profit research hospital 2 (RH2)

The RH2 is a relatively young, private, non-profit hospital established by Italian financial and industrial groups in a major city in Northern Italy during the 1980s. It became an IRCCS in the mid-nineties. The RH2’s mission is to achieve excellence in the field of cancer prevention, diagnosis and treatment through the development of clinical-scientific research and organisational and management innovation, with constant attention to service quality.

In 2003, the RH2 introduced the BSC for two primary reasons. First, the hospital aimed to gain efficiency in response to new public budget constraints enforced by the Lombardy Region, inadequate reimbursements associated with certain DRGs and the saturation of physical resources (such as beds and operating rooms). Second, the BSC was expected to support two important management models implemented in 2002: accreditation to an international quality excellence system and a Human Resources (HR) system aimed at better supporting the professional development of healthcare professionals. The implementation of the BSC was contingent on the presence of a good information system and an organisational culture oriented towards performance.

In 2002, the RH2 developed a pilot area for the building of the BSC by choosing one of the most representative surgical divisions, senology. The logic of the BSC was satisfactorily applied, and the heads of operational units were appreciated for engaging in a performance evaluation that extended beyond just economic aspects.

Since the BSC proved to be an effective tool for communicating strategy and directing individual behaviour, top management extended its use to the whole organisation. This led to the definition of standardised working methods for the team responsible for BSC development, a change management strategy, a cascading methodology to involve all the units of the RH2, the identification of interventions aimed at adapting the information system to the functions required by the BSC, and training and communication initiatives aimed at engaging the staff in the implementation process.

The BSC had a significant impact on the information system. First, it was necessary to ensure the correct data “feeding” of the BSC. Second, the “capillary cascading” (to all the RH2 units) required access to the BSC information for many users.

A software application, Strat&Go, specifically designed for managing BSC was acquired with three main motivations. Firstly, the company wanted managers and professionals to focus on strategic matters based on agreed-upon data, which quickly necessitated the implementation of a centralised data management tool. Secondly, the control meetings needed real-time data analysis to foster a climate of transparency and trust. Finally, managing many performance indicators were costly and required a specific tool.

The BSC progressively qualified as the most ideal location to host the “official” data related to the management of the RH2. Whether Strat&Go is then used to facilitate communication and transparency or another software, this depends on the moment and is not the key aspect (RH2, former CEO).

The MCU involved the hospital IT department in adapting the software application to the organisation’s needs. Therefore, the controller served as a project manager with a purely strategic vision, facilitating the design and implementation of the tool. In this regard, the CEO stated, “*It is important to assume the role of facilitator rather than controller*”.

The BSC was structured on three levels: *Corporate*, which focused on the performance of the organisation as a whole; *Area*, which drilled down into the performance in five homogeneous areas of activity: surgical, medical, services, research and staff; *Organisational unit*, which articulated objectives assigned according to each unit’s characteristics and potential.

In the RH2 experience, the progressive definition of the logical structure of the BSC (perspectives, KPAs, KPIs, cause-and-effect relations) focused on three fundamental aspects: (a) A “capillary” cascading architecture, with all organisational units involved in translating the corporate strategy map into different clinical and research areas handed over to clinical directorates and staff units; (b) A customisation of the logic structure of the BSC, with each operational unit engaged in the selection of KPAs and KPIs and (c) A broad involvement of collaborators from all the professional families (doctors, nurses, researchers, etc.), all required to use the BSC.

The customisation of the BSC logic structure aimed to enhance stakeholders’ perceptions of the multidimensionality and transparency of the tool, contrasting the idea of a purely financial control system. The definition of the BSC logic structure was achieved through meetings (BSC workshops) with active participation from the team responsible for BSC development, including RH2 top management and the heads of the organisational units, along with their closest collaborators. The BSC workshops aimed to identify the key priorities and build a strategy map that highlights cause-and-effect relationships. This was further linked to a control and reporting system, but the logical progression was to strategically launch initiatives that are coherent, with the controller assuming the role of a facilitator.

The BSC is not to be considered as a mere control system, as it is first and foremost a tool that gives its user mental order. It helps understand and strategically analyse the different levels of importance of each task of every day’s life while sorting them out in a way that allows people to focus first on the basics, the things that cannot be done wrong or left behind (RH2, former CEO).

In contrast to the four traditional perspectives, the RH2 created a “slightly more tailored suit” with respect to its structure and the healthcare context. Therefore, the RH2 identified five perspectives: *institutional, research, clinic, teaching, renewal and development* (see Table 1). The “institutional” perspective focuses on evaluating RH2’s ability to effectively interpret its institutional mandate, which includes generating innovation through research, utilising research to benefit patients, ensuring excellence in patient health protection and establishing itself as a school capable of attracting and training the best professional resources. Therefore, RH2 included three further specialised perspectives to explain the results achieved in this regard. The “renewal and development” perspective (referring to the growth and learning traditional perspective of the BSC) focuses on the hospital’s capacity to invest in the future. This involves providing professionals and researchers with cutting-edge technologies, enhancing their skills and creating an environment where RH2 is considered “a great place to work”.

The BSC perspectives remained unchanged until 2012, when the tool was discontinued and the CEO’s term of office ended. The new strategic apex adopted a different management style and focused more on economic results. This led to the adoption of classic management control systems with less emphasis on multidimensionality.

Table 1 shows the evolution of the RH1’s and RH2’s BSC perspectives in comparison with Kaplan and Norton’s (1992) framework.

## Discussion

Based on the analysis conducted, the reasons for the developmental path of a BSC can be attributed to four conceptual areas. These areas reflect the dimensions identified through the socio-technical approach and have emerged from the analysis of the existing BSC literature. The areas include (a) the role and technical features of the BSC in HOs, (b) the role of the internal sponsor in the implementation and development of the BSC, (c) the IT and the controller’s professional skills deployed to manage increasingly complex data and (d) the cultural and organisational drivers and barriers to the introduction of the BSC in professional bureaucracies such as HOs. Table 2 summarises the key elements of the dimensions analysed in the two case studies.

### Role and characteristics of the BSC in the two HOs

The analysis of the two case studies revealed differences in the process of creating the logical structure of the BSC and, consequently, in its main role/function in the two hospitals.

RH1 adopted a unified BSC structure for the entire hospital and all professional staff. However, the tool included a section that identified the different responsibilities of three professional figures for each indicator: clinical unit chiefs, nursing chiefs and health staff. In contrast, RH2 involved all organisational units in the BSC design to customise the logical structure of the tool at the organisational unit level. Therefore, all operators and professional families actively participated in determining KPAs and KPIs, adapting them to their specific needs. The BSC permeates all corporate systems in two different ways, fostering coherence and transparency. An analysis of experts’ opinions confirms the significance of this aspect.

The examination of the two case studies revealed two further differences concerning the use of strategy maps and the creation of cause-and-effect correlations between phenomena. These are two fundamental implementation aspects that stimulate the involved actors to consider linking their activities to those of other organisational units in a systemic logic. RH1 implicitly established cause-and-effect relationships by setting cross-unit objectives with shared KPIs for different organisational units. For example, cost reductions in diagnostics for the laboratory and lowering diagnostic costs per DRG in clinical units led to improvements in clinical and operational processes. Instead, RH2 explicitly identified the cause-and-effect relationships in advance, building the strategy maps and linking the different KPAs of the BSC. RH2 did not test the cause-effect relationships defined in the strategy maps by collecting historical data to run econometric models, but it preserved the articulation of perspectives and KPAs over time.

In each case, the BSC logical framework and the tool were used to serve a primary purpose. In RH1, the tool aimed to monitor performance by evaluating appropriateness, effectiveness and efficiency. Therefore, the BSC aimed to achieve business results by monitoring the performance of each perspective and assessing the extent to which each organisational unit contributed to the overall performance. More specifically, the BSC was intended to ensure transparency in both the budget negotiation phase and the evaluation of objective achievement. This approach is more consistent with the use of the BSC as a performance measurement and control tool (Kaplan and Norton, 1992; Merchant and van der Stede, 2017) and, therefore, useful for monitoring and attention-focusing purposes (Henri, 2006). In contrast, in RH2, the BSC was integrated into the strategic management system (Anthony and Govindarajan, 2014; Atkinson, 2006; Kaplan and Norton, 1996, 2001b, c; Simons, 2014). The aim was to communicate the strategy across the organisation, engaging all operators and professional groups to ensure everyone understood the importance of their role in achieving the strategic goals through actionable steps. Moreover, the BSC served as a means to foster participatory strategic initiatives. This approach is more consistent with a strategic decision-making purpose (Henri, 2006) and coherent with the development of a strategic control tool. This finding contradicts much of the empirical literature, which has found that BSC tools were not originally designed to be used as a strategic management system but rather as management by objective (MBO) mechanisms or merely as information systems (Malmi, 2001). Some authors argue that BSCs are used to generate information to gain support from key stakeholders and obtain legitimacy, especially in the public sector (Bassani *et al*., 2022; Chang, 2007).

### Role of the internal sponsor and top management commitment

From the two cases, it is clear that the strategic apex’s commitment to implementing the control tool and the presence of a leader who fully believes in the BSC project is essential. This prerequisite was positively observed in both case studies, although leadership was assumed by two individuals with different positions in the organisational hierarchy.

Ultimately, the success of any control system depends mainly on its acceptance and dissemination (de Waal, 2002). However, in the healthcare sector, health professionals carry significant influence in corporate governance and are central to any relevant process. Therefore, their alliance with the top management is crucial to any major change.

In RH1, since the beginning of the project, the strategic apex has been committed to providing the MCU with relevant degrees of freedom in the adoption and management phases. Additionally, the consistent presence of the director of the MCU ensured ongoing utilisation and improvement of the BSC, coherently with internal needs and external requests (e.g. Brunetta Law and Liguria Region performance requests). This also nurtured trust in the guidance of the process from the perspective of the organisational units. This continuous, supportive innovation has stimulated the engagement of health professionals in the control process and has fostered the learning process related to the BSC.

At RH2, two significant factors have positively contributed to the introduction of the BSC since the early 2000s: (a) the strong commitment of the CEO, who was the main internal sponsor and in charge of the BSC project and (b) the willingness of the internal stakeholders to support the introduction of the tool.

However, while the BSC tool is still used at RH1, it was abandoned at RH2 in 2012. These two different outcomes have a major underlying motivation. While RH1’s project was continuously supported by the same internal sponsor, in RH2, the BSC was dismissed in conjunction with the departure of the internal sponsor and the arrival of a new CEO with a different management style (more focused on financial metrics).

### IT and controller skills

The computing technology designed to elaborate on the large volume of complex organisational data was one of the most important drivers for the development of the BSC in the two HOs. As a result of such development, the HO could efficiently organise the information flowing from multiple non-aligned data subsystems. The way the implementation process was conducted appears to have made a difference.

The RH1 gradually transformed its information system, starting with the existing technologies and postponing the creation of a data warehouse. The intranet was used to create a management dashboard that allows for drilling down into data at the level of the operating units.

In RH2, the implementation introduces new technological solutions that transform the business information system, especially the existing data warehouse and the intranet, providing all users with easy access to the BSC.

RH1 developed new IT solutions internally, thanks to the MCU, as the director himself has IT and coding skills. This drove the improvement of the MCU’s professional skills and autonomy to develop customised BSC dashboards and databases. The RH2 case showed a different approach that mainly relied on third-party solutions, which were fine-tuned with the hospital’s existing technologies through the information system unit. However, in RH2, the MCU lacked technological leadership in the project.

Therefore, it seems crucial for the MCU to acquire technological skills and autonomy in relation to the BSC for the long-term success of the control tool.

### Professional culture and managerial control

The professional culture within the HO can hinder the implementation of the BSC. This is due to the competing values embedded in the logic of management and professionalism. The two hospitals employed different strategies to engage clinicians with the purpose of having the project accepted and understood by internal stakeholders, ultimately aiming to make the BSC successful.

In RH1, the director of the MCU is also a physician (Director of Occupational Medicine). This was a facilitating factor because health professionals “see a colleague” in the head of management control and project leader; he “speaks their language”, and they can also easily discuss strictly medical matters with him. The MCU has always been committed to including all the heads of specialty units in the process of creating objectives, discussing their feasibility and modifying them whenever deemed necessary. Moreover, RH1 initially introduced the BSC as an experimental tool without any economic leverage, and it only became an official tool for performance evaluation eight years later. During this period, clinicians could familiarise themselves with the tool and build trust in its potential for learning. Even after the BSC became the official evaluation system, clinicians valued the fact that strategic goals were always discussed together with the MCU, and they appreciated the transparency experienced during budget meetings.

Instead, in the second case, the BSC’s adoption was initially favoured by the fact that RH2 was a young organisation with youthful personnel and was experiencing a period of business growth and important projects. These conditions were all favourable for innovation. In this context, managers involved health professionals in the BSC design process to raise their awareness of the BSC’s value. In fact, the perspectives and KPIs were developed in collaboration with the heads of all specialty units, thereby fostering their links with the clinical and research realms. This fostered project transparency, and the actors perceived the BSC as a useful tool that facilitated their practice. This is consistent with the idea that engaging in a participatory performance process may enhance the self-efficacy of hybrid professionals and, indirectly, their capacity to contribute to performance (Giacomelli, 2020; Macinati *et al*., 2016). However, RH2 introduced the BSC as an official evaluation tool for monetary incentives, initially implementing it in a pilot unit and later extending it to all organisational units. This decision may have generated a conflict between the learning potential for strategy-making and the evaluation purposes accompanied by incentives, emphasising the role of control mechanisms. As reported in the literature, this situation may be conducive to biases, as professionals may provide distorted information when defining performance goals and targets to increase the probability of receiving a favourable evaluation (Simons, 2014).

## Conclusions

The study yielded interesting results regarding the application of an integrated and multidimensional control tool, such as the BSC, in professional and complex organisations.

We analysed only two cases. However, three aspects support the validity of our interpretations. First, both organisations have been pioneers in the introduction of BSC. Therefore, we can analyse the different stages of the evolution of this tool and understand the structural and practical reasons that influence corporate decisions. This is consistent with the socio-technical approach, which assumes that an organisation continues to change and that the implementation of innovation “is not immediate and several transition states may be passed through by the organization during the implementation process” (Bostrom and Heinen, 1977a, p. 28). Second, our interpretations apply to both public and private HOs. Finally, the interviewees were privileged observers and key actors in the analysed organisations.

The results highlighted different factors that drive or hinder the effective implementation and evolution of the BSC.

First, top management commitment and a strong internal sponsor seem to be necessary conditions for achieving successful and consistent implementation of the BSC. This is consistent with the analysis by Kaplan and Norton (2001a), which defines leadership as the most important variable expressing the success or failure of BSC continuity. The transition of the people who are the main sponsors of the BSC, also due to mergers of organisations, may determine the dismissal of the tool. Moreover, staff groups or functional officers may introduce the BSC, but the lack of commitment and personal involvement of the CEO and senior management in the development process can cause its failure (Inamdar *et al*., 2002; Kaplan and Norton, 2001a). The development of a formal information system as sophisticated as the BSC requires: (a) specific skills and competencies to maintain the system successfully and (b) a significant amount of time to involve the whole organisation and align efforts. If the internal sponsor is missing during the implementation of the BSC and its use as an official (organisation-wide) management tool, the likely result is that the BSC will collapse. Moreover, it is equally important, especially in the early stages, to have strong commitment and support from the strategic apex to enable the internal sponsor to proceed with the project. This aspect is extremely relevant for public HOs where the turnover of general managers is particularly high, averaging about 3.5 years (Cinelli *et al*., 2020).

Second, the presence of clear and formalised directional strategies, including mission statements, is of paramount importance. Both RH1 and RH2 have missions that aim to pursue excellence in different performance areas (such as research, clinical, organisational and managerial), and this can be considered a driver for the implementation of a multidimensional control system. One significant point to consider is the necessity of adapting the BSC’s perspectives to meet the specific missions of HOs. Moreover, teaching and research hospitals are particularly complex organisations with a threefold mission (Carbone *et al*., 2010; Smith and Whitchurch, 2002): (1) the training and specialisation of future doctors, (2) the development of scientific research and (3) the provision of specialised and innovative healthcare. Therefore, it is important for the BSC to address all three missions by introducing the research perspective and the teaching perspective. A clear difference has emerged between businesses and HOs in terms of the economic and financial aspects and the corresponding KPIs included in the BSC. While these aspects are extremely relevant in the BSC for businesses, they are less emphasised in the case of HOs. This trend is evident not only in public HOs but also in private ones, as indicated by the analysis studies.

Third, the presence of an integrated IT system and specific software for measuring healthcare performance is a favourable factor for developing the BSC as a means to manage the complexity of the instrument due to the elevated number of KPIs and to ensure the quality and accuracy of data and information. Nevertheless, information technologies by themselves do not automatically make these systems perceived as useful. Expertise is required to keep them constantly aligned with external information requests and internal organisational needs.

In contrast, a hindering factor involves professionals who “do not like to be measured”. Three characteristics of BSC (that make the tool more suitable for the professional culture) can help to deal with the aforementioned barriers: (1) It is a multidimensional system that not only analyses classic economic and financial data but also extends its analytical power to different operating areas (e.g. clinical and research). (2) Users can identify cause-and-effect correlations between various indicators. (3) Users can perceive the transparency of the measurement system. A strong and talented managerial control staff is crucial for fostering a culture of trust in hospitals, where there tends to be a bit of a “chronic wall” between the administration and the clinical staff. The need to constantly monitor and interpret performance from both an economic and clinical viewpoint helped to mitigate the traditional contrast between the administrative and professional components of the organisational culture. This made each side more aware of the problems and needs of the other, fostering mutual understanding. An additional point of strength concerns the fact that the BSC communicates the strategy and involves everyone more actively in achieving it. In fact, each organisational member can understand the importance of their role within a defined path for achieving corporate goals, so they know what they must do to personally contribute to the achievement of the overall objectives. Therefore, it improves the way people work.

Finally, the two case studies demonstrated that the BSC can coherently internalise objectives set by external constituencies, as evidenced in the RH1 for the goals/controls established by the Liguria Region, PNE, etc. This is a relevant point for public organisations because they are easily permeable to institutional and strategic changes mandated by government authorities. In addition, and linked to the previous point, the BSC is resilient in separating strategy from control. This is particularly important for organisations that face challenges in defining a strategy due to the constraints imposed by healthcare institutional settings.

Moreover, it is important to highlight the limitations of the study and, consequently, suggest avenues for further research.

First, we adopted a longitudinal perspective but with a retrospective approach, which may introduce memory bias. However, the duration of the observation period in this study poses significant challenges for employing a prospective approach.

Second, the data collection relied on interviews with the two managers responsible for the design, introduction and development of the BSC in the two organisations from the outset. However, no other subjects from the two HOs participated in the project throughout the entire observation period of the two cases.

Further developments in research may lead to an increase in the number of interviewees with specific research aims, which may justify focusing on segments of the observation period. For instance, incorporating multiple professional profiles could assist in analysing the role of interprofessional relationships in the use/implementation of the BSC. Finally, increasing the number of case studies, especially from different national contexts, may improve the evidence supporting the elements of the results in the four conceptual areas: (1) the role and technical features of the BSC in HOs, (2) the role of the internal sponsor in the implementation and development of the BSC, (3) the IT and the controller’s professional skills deployed to manage increasingly complex data and (4) the cultural and organisational drivers and barriers to the introduction of the BSC in professional bureaucracies such as HOs.

## Figures and Tables

**Table 1:** A comparison of the BSC perspectives in the two case studies

Classic perspectives Kaplan and Norton (1992)	Case 1: Public research hospital (RH1)			Case 2: Private non-profit research hospital (RH2)
	2004	2012	2021	2003
1. Financial	1. Costs	1. Resources	1. Resources	1. Institutional
2. Internal processes	2. Production	2. Activities	2. Activities	2. Research
3. Customers	3. Efficiency	3. Organisation	3. Organisation	3. Clinic
4. Learning and growth	4. Organisation and quality	4. Quality, security and risk control	4. Quality, security and risk control	4. Teaching
		5. Research	5. Research	5. Renewal and development
			6. Appropriateness	
			7. Effectiveness	
			8. Efficiency	

**Source(s):** Authors’ work

**Table 2:** Comparative summary of the key factors in the two case studies

Dimensions	Key factors	RH1	RH2
Role and characteristics of the BSC in the two HOs	Adjusted Perspectives	Limited focus on the “economic”perspective: efficiency and cost indicators scattered in different perspectives. Introduction of the research perspective in 2012	Limited focus on the “economic” perspective: efficiency and cost indicators scattered in different perspectives. Introduction of the research and the teaching perspectives
	Cause-and-Effect Relationship	No use of strategy maps. Cause-and-effect relations embedded in cross-units’ objectives/KPIs	Use of strategy maps. E*x ante* identification of cause-and-effect relations, but no method used to test them ex-post
	Underlying Logic	Performance measurement and monitoring	Strategy communication and translation into action
Role of the internal sponsor and top management commitment	Internal Sponsor	Director of Management Control Continuity in the role since 2002	CEO and project manager for BSC. Controller as “right-hand man” and operational part of the project
	Top Management Commitment	Essential for “start-up” and ongoing development. Instrument are “defended” in periods of lack of commitment	Coincidence of the roles of internal sponsor and CEO. Abandonment of the instrument at the exit of the internal sponsor in 2013
Information technology and controller’s skills	Information Technology	Necessary driver for the introduction and development of BSCs, which require information and data from different sub-systems. Use and development of existing software packages	Necessary driver for the introduction and development of BSCs, which require information and data from different sub-systems. Use of Strat&Go package built by Kaplan and Norton for BSC management
	Controller’s Skills and Support	Controller’s IT programming skills. Management and development of databases and software are in the hands of the Management Control unit	Controller, in addition to his classical role, became a facilitator of strategic development processes. The ICT unit involved in adapting the existing applications to the BSC needs
Professional culture and managerial control	Clinicians’ Engagement	DMC with a medical background can be a key part of both clinical and technical discussions. Continuity and transparency inspire trust. Heads of clinical units involved in discussions about the relevance and feasibility of objectives/targets	DMC role of process facilitator. BSC is defined as a means of transparency on multidimensional aspects of performance. Clinical involvement in the definition of KPAs and KPIs. In 2002, RH2 was a young and growing company with a spirit of innovation in both clinical and managerial matters

**Source(s):** Authors’ work
